# Stainless Steel Versus Titanium Miniplates in the Treatment of Mandibular Fractures: A Comparative Study

**DOI:** 10.7759/cureus.79912

**Published:** 2025-03-02

**Authors:** Saloni Bharti

**Affiliations:** 1 Dentistry, Employees' State Insurance Corporation (ESIC) Medical College and Hospital, Patna, IND

**Keywords:** intermaxillary fixation (imf), mandibular fracture, maxillomandibular fixation (mmf), open reduction and internal fixation (orif), stainless steel, titanium miniplates

## Abstract

Introduction

The fast-paced lifestyle of modern society has led to an increase in maxillofacial trauma, particularly in the mandible, which is prone to fracture due to its prominent position among facial bones. Mandibular fractures are one of the most common facial injuries and require effective treatment to ensure proper healing and function. A study conducted at the Department of Oral and Maxillofacial Surgery of Buddha Institute of Dental Sciences and Hospital investigated the adaptability, impact on fracture line difficulties, and facial outcomes of fracture therapy using stainless steel and titanium miniplates. The research aimed to compare these two materials in terms of their effectiveness, patient outcomes, and any complications that might arise from their use.

Methodology

The study included 20 adult patients with mandibular fractures, divided into two groups for open reduction and internal fixation using either titanium or stainless steel miniplates. All patients underwent comprehensive diagnostics, including blood tests and virus screenings, prior to the operation to ensure they were suitable candidates for surgery. The surgical procedure was followed by a meticulous postoperative care plan, which included a soft diet to minimize strain on the jaw, chlorhexidine mouth rinse to maintain oral hygiene, and guided elastics to support proper alignment during healing. The study meticulously recorded the operating times, fracture stability, and any complications to provide a thorough comparison between the two materials.

Results

The results showed that patients in the titanium miniplate group had significantly shorter operating times, averaging 32 minutes compared to 45.10 minutes in the stainless steel group (p < 0.0001). This difference highlights the efficiency of titanium plates in surgical procedures. Additionally, using a bimanual examination to assess fracture stability, the titanium plate group exhibited superior stability compared to the stainless steel plate group. The stability of the fractured segments in the titanium group was statistically significant with a p-value of 0.474.

Conclusion

Titanium miniplates provide several advantages over stainless steel in the treatment of mandibular fractures. These advantages include increased postoperative stability, lower complication rates, and higher biocompatibility. The study concludes that titanium miniplates are a more effective option for mandibular fracture treatment, offering better patient outcomes and reducing the overall burden on healthcare resources. This finding is significant for oral and maxillofacial surgery, as it supports the use of titanium miniplates for improved surgical results and patient care.

## Introduction

The fast-paced, result-oriented lifestyle of modern society has led to an increase in various forms of accidents and violence, resulting in a notable rise in maxillofacial trauma. Among the facial bones, the mandible is particularly susceptible to fractures due to its prominent position [1,2]. Mandibular fractures, especially at the symphysis or parasymphysis, account for approximately 20% of facial bone injuries. Common causes of mandibular fractures include interpersonal violence, falls, sports injuries, and industrial accidents [3].

Dental surgeons frequently encounter mandibular fractures, which can also result from tooth extractions, especially when impacted third molars are involved. Treatment approaches for these fractures have significantly evolved over the last 30 years, transitioning from wire-based techniques and jaw fixation to open reduction and internal fixation (ORIF) using miniplates [4,5]. This shift has improved outcomes by reducing complications such as malocclusion, non-union, and limited mouth opening while enhancing patient comfort and recovery time. Previously, mandibular fractures were managed with closed reduction techniques like inter-dental wiring and maxillomandibular fixation, which, despite being effective, posed several disadvantages, including patient discomfort, airway obstruction, and prolonged recovery periods [6,7]. In contrast, modern methods favor ORIF, which allows for immediate functional recovery. Miniplates, especially titanium ones, have become standard due to their reliability and functional stability. Additionally, several metals, including gold, silver, copper and its alloys, lead, and aluminum and its alloys, were used and tested, while stainless steel emerged through the era as the new corrosion-resistant material. However, titanium claims lots of advantages over classic stainless steel. Titanium is widely used in the production of dental and orthopedic implants because of its excellent biocompatibility, which refers to a material's ability to function well within a specific application and elicit an appropriate response from the host [8-10].

In this study, a comparative study of fixation with stainless steel miniplates and titanium miniplates was undertaken for fractures of the mandible. The study focused on the adaptability, impact on fracture line complications, and facial outcomes of fracture management at the Department of Oral and Maxillofacial Surgery of Buddha Institute of Dental Sciences and Hospital.

## Materials and methods

Study design and setting

This comparative study was conducted with 20 patients from the Department of Oral and Maxillofacial Surgery of Buddha Institute of Dental Sciences and Hospital in Patna, India, after obtaining ethical approval from the Institutional Ethics Committee of Employees' State Insurance Corporation (ESIC) Medical College and Hospital (approval number: IEC/ESIC-MC-Bihta/2024/002) and following the guidelines outlined in the Declaration of Helsinki. Patients with fractures of the mandible at different anatomical locations were randomly divided into two groups for a surgical procedure called ORIF using miniplates. Group 1 received titanium miniplates, whereas group 2 received stainless steel miniplates. The plates and screws were of standard design, size, and calibration and metallic composition (ORTHOMAX, Burwood, Australia). These miniplates have a thickness of 2 mm, with four non-locking holes, and are designed for adaptation using 8-mm-long screws. The effectiveness of the treatment was compared between the two groups.

Selection Criteria

Patients were subjected to clinical and radiographic assessment using standard mandible radiographs (orthopantomogram (OPG) and posteroanterior (PA) view). Subsequently, they were scheduled for surgery under general anesthesia after regular preoperative investigations and evaluations. The inclusion criteria consisted of adult patients with different forms of mandibular fractures, except for condyle fractures, non-united fractures, mal-united fractures, and comminuted fractures, and individuals with medical conditions such as diabetes, with smoking habits, with age below 16, and receiving steroids.

Data sources and variables 

Surgical Procedures

**Figure 1:**
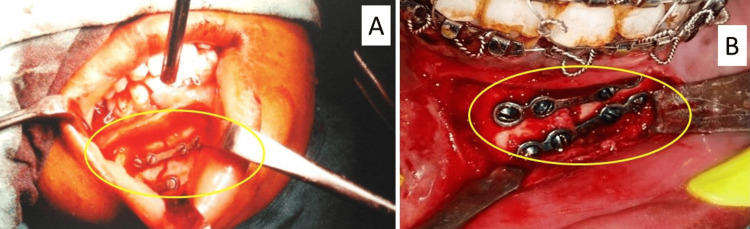
Placement of plates Panel A shows a stainless steel miniplate. Panel B shows a titanium miniplate

Patients who were under general anesthesia underwent surgical procedures that included making incisions in the intraoral vestibular area, dissecting the tissue below the periosteum, and utilizing 2 mm miniplates to fixate the bones according to Champy's principles. The plates were carefully placed to prevent nerve damage and assure stability which can be seen in Figure 1.

Postoperative Care

The patient will be instructed to follow a diet consisting of soft foods, use a chlorhexidine mouth rinse, and use guided elastics to maintain proper occlusion. Follow-up assessments will be scheduled at the third day, eighth day, first month, and third month to assess the progress of healing and monitor for any complications such as pain, swelling, infection, and paresthesia. The armamentarium for the procedures included standard surgical instruments and fixation devices, ensuring uniformity in treatment across both groups.

Statistical analysis

Data entry was done using Excel spreadsheets (Microsoft Corp., Redmond, WA, USA). Numerical data were analyzed using Student's t-test, and categorical data were analyzed using Fisher's exact test and chi-squared test. Statistical analysis was carried out using the Statistical Package for Social Sciences (SPSS) program version 15 (SPSS Inc., Chicago, IL, USA) and GraphPad software version 5 (Insight Venture Management, LLC, New York, NY, USA). A p-value of ≤0.05 indicated statistical significance.

## Results

Table 1 presents the gender-wise distribution of subjects across different materials. For the titanium miniplate, there were five women (50%) and five men (50%). In the stainless steel miniplate group, all 10 subjects were men (100%). Overall, five women (25%) and 15 men (75%) were included in the study, with a total of 20 subjects across both materials.

**Table 1:** Gender-wise distribution of subjects

Gender	Miniplate				Total	
	Titanium		Stainless steel			
	Frequency (n)	Percentage (%)	Frequency (n)	Percentage (%)	Frequency (n)	Percentage (%)
Female	5	50%	0	0%	5	25%
Male	5	50%	10	100%	15	75%
Total	10	100%	10	100%	20	100%

Table 2 shows the fracture types among the 20 subjects. The most common was left parasymphysis fracture (35%), with four cases treated with titanium and three with stainless steel. Right parasymphysis fractures were 15%, all treated with titanium. Symphysis fractures were 15%, with one treated with titanium and two with stainless steel. Other fracture types, including angle, body, and combined fractures, each accounted for 5-10% of cases. Both titanium and stainless steel groups had 10 fractures each.

**Table 2:** Distribution of fracture within the subjects

Type of fracture	Miniplate				Total	
	Titanium		Stainless steel			
	Frequency (n)	Percentage (%)	Frequency (n)	Percentage (%)	Frequency (n)	Percentage (%)
Angle fracture	0	0%	1	10%	1	5%
Left body fracture	0	0%	1	10%	1	5%
Left parasymphysis and right angle fracture	0	0%	2	20%	2	10%
Left parasymphysis fracture	4	40%	3	30%	7	35%
Right and left parasymphysis fracture	1	10%	0	0%	1	5%
Right body fracture	0	0%	1	10%	1	5%
Right parasymphysis fracture	4	40%	0	0%	4	20%
Symphysis fracture	1	10%	2	20%	3	15%
Total	10	100%	10	100%	20	100%

Table 3 compares the mean time of the procedure between titanium and stainless steel miniplates. The mean procedure time for titanium was 32 minutes (SD = 4.35), while for stainless steel, it was 45.10 minutes (SD = 6.19). The difference was statistically significant with a p-value of <0.0001.

**Table 3:** Comparison of time of procedure within the subjects Significant (s) SD: standard deviation

Time of procedure (min)	Titanium miniplate		Stainless steel miniplate		Student's t-test (p-value)
	Mean	SD	Mean	SD	<0.0001 (s)
	32	4.35	45.10	6.19	

Table 4 shows the distribution and comparison of the stability of fracture segments at the third day between subjects with titanium and stainless steel miniplates. In the titanium group, all 10 subjects (100%) had good stability. In the stainless steel group, eight subjects (80%) had good stability, while two subjects (20%) had fair stability. Overall, out of 20 subjects, 18 (90%) had good stability, and two (10%) had fair stability. Fisher's exact test yielded a p-value of 0.474, indicating a non-significant difference between the groups.

**Table 4:** Distribution and comparison of stability of fracture at the third day of visit within the subjects Fisher's exact test, p-value = 0.474 (s); significant (s); non-significant (ns)

Stability of fracture segment (third day)	Miniplate				Total	
	Titanium		Stainless steel			
	Frequency (n)	Percentage (%)	Frequency (n)	Percentage (%)	Frequency (n)	Percentage (%)
Fair	0	0%	2	20%	2	10%
Good	10	100%	8	80%	18	90%
Total	10	100%	10	100%	20	100%

Table 5 details the occlusion status of subjects on the third day post-procedure. Among those treated with titanium miniplates, all 10 patients (100%) had pre-trauma occlusion. In the stainless steel group, eight patients (80%) had pre-trauma occlusion, while one patient (10%) experienced a mild discrepancy, and another patient (10%) had a moderate discrepancy. Overall, 90% of the subjects had pre-trauma occlusion, and 5% had either a mild or moderate discrepancy. The chi-squared test resulted in a value of 2.222 with 2 degrees of freedom and a p-value of 0.329, indicating the findings were not statistically significant.

**Table 5:** Distribution and comparison of occlusion at the third day of visit within the subjects Chi-squared value = 2.222; degrees of freedom = 2; p-value = 0.329 (ns); significant (s); non-significant (ns)

Occlusion (third day)	Miniplate				Total	
	Titanium		Stainless steel			
	Frequency (n)	Percentage (%)	Frequency (n)	Percentage (%)	Frequency (n)	Percentage (%)
Mild discrepancy	0	0%	1	10%	1	5%
Moderate discrepancy	0	0%	1	10%	1	5%
Pre-trauma occlusion	10	100%	8	80%	18	90%
Total	10	100%	10	100%	20	100%

Table 6 shows the distribution and comparison of pain at the eighth day between subjects with titanium and stainless steel miniplates. In both groups, nine subjects (90%) had no pain, and one subject (10%) experienced mild pain. Overall, out of 20 subjects, 18 (90%) had no pain, and two (10%) experienced mild pain. Fisher's exact test yielded a p-value of 1.00, indicating a non-significant difference between the groups.

**Table 6:** Distribution and comparison of pain at the eighth day of visit within the subjects Fisher's exact test, p-value = 1.00 (ns); significant (s); non-significant (ns)

Pain (eighth day)	Miniplate				Total	
	Titanium		Stainless steel			
	Frequency (n)	Percentage (%)	Frequency (n)	Percentage (%)	Frequency (n)	Percentage (%)
Absent	9	90%	9	90%	18	90%
Mild	1	10%	1	10%	2	10%
Total	10	100%	10	100%	20	100%

Table 7 shows the distribution and comparison of swelling at the eighth day between subjects with titanium and stainless steel miniplates. In the titanium group, all 10 subjects (100%) had no swelling. In the stainless steel group, nine subjects (90%) had no swelling, while one subject (10%) had moderate swelling. Overall, out of 20 subjects, 19 (95%) had no swelling, and one (5%) had moderate swelling. Fisher's exact test yielded a p-value of 1.00, indicating a non-significant difference between the groups.

**Table 7:** Distribution and comparison of swelling at the eighth day of visit within the subjects Fisher's exact test, p-value = 1.00 (ns); significant (s); non-significant (ns)

Swelling (eighth day)	Miniplate				Total	
	Titanium		Stainless steel			
	Frequency (n)	Percentage (%)	Frequency (n)	Percentage (%)	Frequency (n)	Percentage (%)
Absent	10	90%	9	90%	19	95%
Moderate	0	10%	1	10%	1	5%
Total	10	100%	10	100%	20	100%

Table 8 shows the distribution and comparison of mouth opening between titanium and stainless steel groups at different intervals: the third day, the eighth day, the first month, and the third month. On the third day, the mean mouth opening was significantly higher for the titanium group (32.10 ± 2.18) compared to the stainless steel group (24.10 ± 2.42), with a p-value of <0.0001. By the eighth day, the difference was not significant, with means of 35.00 ± 3.23 for titanium and 32.10 ± 3.93 for stainless steel (p = 0.0882). At the first month, the means were 36.10 ± 3.28 for titanium and 33.90 ± 2.64 for stainless steel (p = 0.1161). By the third month, the means were 38.30 ± 2.87 for titanium and 36.90 ± 4.68 for stainless steel (p = 0.4303). The results indicate significant differences only at the third-day interval.

**Table 8:** Distribution and comparison of mouth opening within the subjects at the third day, eighth day, first month, and third month of visit SD: standard deviation Significant (s); non-significant (ns)

Mouth opening	Titanium		Stainless steel		Student's t-test (p-value)
	Mean	SD	Mean	SD	
Third day	32.10	2.18	24.10	2.42	<0.0001 (s)
Eighth day	35.00	3.23	32.10	3.93	0.0882 (ns)
First month	36.10	3.28	33.90	2.64	0.1161 (ns)
Third month	38.30	2.87	36.90	4.68	0.4303 (ns)

Table 9 shows the distribution and comparison of wound dehiscence at the eighth day between subjects with titanium and stainless steel miniplates. In the titanium group, all 10 subjects (100%) had no wound dehiscence. In the stainless steel group, nine subjects (90%) had no wound dehiscence, while one subject (10%) had wound dehiscence. Overall, out of 20 subjects, 19 (95%) had no wound dehiscence, and one (5%) had wound dehiscence. Fisher's exact test yielded a p-value of 1.00, indicating a non-significant difference between the groups.

**Table 9:** Distribution and comparison of wound dehiscence at the eighth day of visit within the subjects Fisher's exact test, p-value = 1.00 (ns); significant (s); non-significant (ns)

Wound dehiscence (eighth day)	Miniplate				Total	
	Titanium		Stainless steel			
	Frequency (n)	Percentage (%)	Frequency (n)	Percentage (%)	Frequency (n)	Percentage (%)
Absent	10	100%	9	90%	19	95%
Present	0	0%	1	10%	1	5%
Total	10	100%	10	100%	20	100%

Table 10 shows the distribution and comparison of infection at the first month between subjects with titanium and stainless steel miniplates. In the titanium group, all 10 subjects (100%) had no infection. In the stainless steel group, eight subjects (80%) had no infection, while two subjects (20%) had an infection. Overall, out of 20 subjects, 18 (90%) had no infection, and two (10%) had an infection. Fisher's exact test yielded a p-value of 0.474, indicating a non-significant difference between the groups.

**Table 10:** Distribution and comparison of infection at the first month of visit within the subjects Fisher's exact test, p-value = 0.474 (ns); significant (s); non-significant (ns)

Infection (first month)	Miniplate				Total	
	Titanium		Stainless steel			
	Frequency (n)	Percentage (%)	Frequency (n)	Percentage (%)	Frequency (n)	Percentage (%)
Absent	10	100%	8	80%	18	90%
Present	0	0%	2	20%	2	10%
Total	10	100%	10	100%	20	100%

Table 11 shows the distribution and comparison of paresthesia at the third day between subjects with titanium and stainless steel miniplates. In the titanium group, nine subjects (90%) had no paresthesia, while one subject (10%) experienced paresthesia. In the stainless steel group, eight subjects (80%) had no paresthesia, and two subjects (20%) experienced paresthesia. Overall, out of 20 subjects, 17 (85%) had no paresthesia, and three (15%) experienced paresthesia. Fisher's exact test yielded a p-value of 1.00, indicating a non-significant difference between the groups.

**Table 11:** Distribution and comparison of paresthesia at the third day of visit within the subjects Fisher's exact test, p-value = 1.00 (ns); significant (s); non-significant (ns)

Paresthesia (third day)	Miniplate				Total	
	Titanium		Stainless steel			
	Frequency (n)	Percentage (%)	Frequency (n)	Percentage (%)	Frequency (n)	Percentage (%)
Absent	9	90%	8	80%	17	85%
Present	1	10%	2	20%	3	15%
Total	10	100%	10	100%	20	100%

## Discussion

Mandible fracture treatment aims to restore both anatomical form and function, with a particular focus on achieving proper occlusion. While closed reduction and intermaxillary fixation (IMF) have historically been effective, there has been a recent trend towards ORIF [11].

Barber et al. and Evenhuis et al. summarized that in recent decades, rigid internal fixation using titanium miniplates and screws has become widely adopted for managing mandible fractures. Titanium's exceptional biocompatibility, characterized by the rapid formation of an oxide film and resistance to corrosion, has contributed to its widespread acceptance. However, the cost of titanium implants can be prohibitive for some patients, particularly those from economically disadvantaged backgrounds [12,13]. Stainless steel, derived from biological-grade stainless steel, has been a longstanding alternative in mandible fracture treatment. Its affordability and documented biocompatibility make it a viable option. Similar to the study of Seyhan et al., this clinical study compared titanium and stainless steel plating systems across various parameters, including postoperative stability, adaptability, surgical time, and the occurrence of postoperative complications, such as sensory deficits, pain, swelling, difficulty in mouth opening, and infection, as well as their impact on occlusion [12,14].

As compared with El-Zayat et al.'s study, in the present study, the duration of surgery, from incision to closure, was compared between the titanium and stainless steel groups. The average surgical time was notably shorter in the titanium group compared to the stainless steel group. Specifically, the average surgical time was 32 minutes in the stainless steel group and 45.10 minutes in the titanium group. The mean time for the procedure with titanium miniplates was 32 minutes, whereas with stainless steel miniplates, it was 45.10 minutes. Statistical analysis revealed a significant difference between the two groups, with a p-value of 0.001. This suggests that titanium plates offer greater adaptability and efficiency during surgery. Studies have shown that titanium miniplates are easier to adapt and require less time for fixation compared to stainless steel plates [15].

As per the Janssen and Kloen study, fracture stability, crucial for successful treatment outcomes, was evaluated in both groups through the manual examination of mobility at the fractured site preoperatively and during follow-up. The present findings indicate significantly better stability in cases treated with titanium plates compared to stainless steel. Several factors contribute to instability, including postoperative infection, surgical experience, and errors during fixation device placement. Infection, for instance, can create a hypoxic environment leading to fibrous union or non-union [16]. Achieving a balance between micro- and macromovement is critical for stimulating vascular ingrowth and ensuring successful treatment.

Al-Hassania et al. focused on considering these factors; IMF was employed postoperatively in cases of instability. Titanium's biomechanical properties, including higher tensile strength, compressive strength, and lower modulus of elasticity, facilitate better adaptation and maintenance of reduced segments, ultimately leading to improved stability. Titanium's inert nature also allows its use in the presence of infection, further enhancing treatment success [17].

In contrast, two cases in the stainless steel group experienced compromised stability due to infection-related complications, including lacerated lingual mucosa and tooth involvement. However, these cases did not require surgical intervention and were managed with aggressive antibiotic therapy and maintenance care, including continuous irrigation. While the p-value for the stability of fractured segments was not statistically significant (0.474), the present findings align with previous studies, demonstrating good stability in most cases, especially in the titanium group.

Hussein et al. established that optimal pre-existing occlusion is paramount to avoid postoperative malocclusion, which can lead to extensive rehabilitation or even necessitate re-surgery. The present evaluation categorized postoperative occlusion into three levels: pre-existing trauma, mild discrepancy, and moderate discrepancy. In the present study, occlusion was harmonious or pre-existing in all patients in group 1, while two patients in group 2 exhibited mild discrepancies, which were managed with elastic assistance or selective grinding. Factors contributing to occlusal discrepancies include patient dental status, fracture characteristics, and the accuracy of reduction and fixation during surgery [18]. The p-value for occlusion was not significant (0.329), and none of the patients required re-surgery. The present findings are consistent with previous research, which also reported similar incidences of occlusal discrepancies and their management through elastic retention or selective grinding.

Chun et al. explained that wound dehiscence post-surgery can result from various factors, including inadequate tissue closure, postoperative infection, and patient negligence in maintaining oral hygiene or adhering to postoperative care instructions. In the present study, two patients in the stainless steel group experienced wound dehiscence, one due to inadequate tissue for proper closure and the other due to postoperative negligence [19]. Both cases were managed with irrigation and antibiotic therapy, with one requiring re-suturing. The absence of wound dehiscence in the titanium group can be attributed to titanium's biocompatibility, facilitating rapid oxide film formation and corrosion resistance, as observed in previous research.

Phillips et al. described that postoperative sensory deficits, or paresthesia, can result from various causes such as nerve entrapment, excessive manipulation during reduction, nerve stretching, or hardware penetration into nerve canals. In the present study, two patients in the stainless steel group experienced sensory deficits, one of which was pre-existing and resolved postoperatively, while the other resulted from hardware penetration. Both cases were managed with methylcobalamin and Neurobion Forte. In the titanium group, one patient experienced minimal lip numbness due to manipulation during surgery, which resolved quickly. The present findings align with previous studies, indicating similar rates of sensory deficits in both groups [20].

Hassan reported that postoperative decreases in mouth opening, or trismus, can result from various factors including swelling, infection, or discomfort. In the present study, mouth opening decreased postoperatively but improved over time, with physiotherapy aiding recovery. The duration of surgery was shorter in the titanium group, resulting in less postoperative swelling compared to the stainless steel group [21]. While one case in the stainless steel group experienced prolonged swelling due to wound dehiscence and infection, timely intervention resolved the issue. The present findings are consistent with previous research advocating postoperative mouth exercises for improved outcomes.

Krishna et al. demonstrated that postoperative swelling and edema are common after surgery, influenced by factors such as surgical duration, tissue manipulation, and fracture characteristics. In the present study, the titanium group experienced minimal swelling compared to the stainless steel group, which had cases of mild to moderate swelling. Timely intervention effectively managed swelling-related complications, highlighting the importance of postoperative care and monitoring [22].

Nikolajsen and Minella studied postoperative pain, measured using the visual analog scale. Accordingly, it varied among patients and was influenced by factors such as fracture severity, wound complications, and surgical duration. While both groups experienced postoperative pain, timely intervention and appropriate management ensured resolution. The present findings align with previous studies reporting similar pain management outcomes [23].

Eraqi et al. explained that postoperative infection risk is influenced by factors such as oral hygiene, patient compliance, and material biocompatibility. In the present study, one patient in the stainless steel group developed an infection due to inadequate tissue closure, while another case was attributed to patient factors. In contrast, the titanium group had no infection cases, highlighting titanium's superior biocompatibility. The current findings support previous research advocating titanium's use in reducing infection risk [24].

Limitations of the study

A small sample size and the single-center design potentially compromise the study's generalizability. Additionally, a brief follow-up period of three months may not capture long-term outcomes adequately. Future research with larger sample sizes, multi-center designs, longer follow-up periods, and comprehensive cost analyses is required to validate these findings and enhance their clinical relevance.

## Conclusions

Titanium miniplates offer significant advantages over stainless steel in the treatment of mandibular fractures, including better postoperative stability, reduced complication rates, and superior biocompatibility. These findings support the continued use and further development of titanium-based fixation systems in maxillofacial surgery.

## References

[1] Passi D, Ram H, Singh G, Malkunje L (2014). Total avulsion of mandible in maxillofacial trauma. Ann Maxillofac Surg.

[2] Halsey JN, Hoppe IC, Granick MS, Lee ES (2017). A single-center review of radiologically diagnosed maxillofacial fractures: etiology and distribution. Craniomaxillofac Trauma Reconstr.

[3] Noor M, Hassan R, Bukhari AH, Hilal R (2022). Frequency of parasymphysis fracture in mandibular fractures due to road traffic accidents. Pak J Med Health Sci.

[4] Singh MM, Rajpal S, Priya N, Ali MG, Akhtar S (2021). CAP splint: an armour to safeguard developing dentition in paediatric mandibular fractures- a case series. Indian J Orthod Dentofacial Res.

[5] Lothamer C, Snyder CJ, Duenwald-Kuehl S, Kloke J, McCabe RP, Vanderby R Jr (2015). Crown preservation of the mandibular first molar tooth impacts the strength and stiffness of three non-invasive jaw fracture repair constructs in dogs. Front Vet Sci.

[6] Lee J, Jung HY, Ryu J, Jung S, Kook MS, Park HJ, Oh HK (2022). Open versus closed treatment for extracapsular fracture of the mandibular condyle. J Korean Assoc Oral Maxillofac Surg.

[7] Akter S, Ashrafi Z, Manira S, Rahman MA, Rashid MH (2023). Postoperative complications after surgical management of mandibular angle fracture. Update Dent Coll J.

[8] Aroussi D, Aour B, Bouaziz AS (2019). A comparative study of 316L stainless steel and a titanium alloy in an aggressive biological medium. Eng Technol Appl Sci Res.

[9] Santos GA (2017). The importance of metallic materials as biomaterials. Adv Tissue Eng Regen Med.

[10] Baltatu MS, Tugui CA, Perju MC, Benchea M, Spataru MC, Sandu AV, Vizureanu P (2019). Biocompatible titanium alloys used in medical applications. Rev Chim (Bucharest).

[11] Ng KB, Liu TH, Lee SS (2024). Three-dimensional navigation-assisted open reduction and internal fixation of a subcondylar and ramus fracture on a maxillary oligodontia patient. J Craniofac Surg.

[12] Barber CC, Burnham M, Ojameruaye O, McKee MD (2021). A systematic review of the use of titanium versus stainless steel implants for fracture fixation. OTA Int.

[13] Evenhuis JV, Verstraete FJ, Arzi B (2022). Management of failed stainless steel implants in the oromaxillofacial region of dogs. Front Vet Sci.

[14] Seyhan M, Guler O, Mahirogullari M, Donmez F, Gereli A, Mutlu S (2018). Complications during removal of stainless steel versus titanium nails used for intramedullary nailing of diaphyseal fractures of the tibia. Ann Med Surg (Lond).

[15] El-Zayat BF, Ruchholtz S, Efe T, Paletta J, Kreslo D, Zettl R (2013). Results of titanium locking plate and stainless steel cerclage wire combination in femoral fractures. Indian J Orthop.

[16] Janssen SJ, Kloen P (2022). Supercutaneous locking compression plate in the treatment of infected non-union and open fracture of the leg. Arch Orthop Trauma Surg.

[17] Al-Hassania ES, Dawood JJ, Al-Sabe'a BM (2019). Compression and wear properties of biocompatible commercially pure titanium and (titanium-silicon) alloys. Anbar J Eng Sci.

[18] Hussein MA, Besher A, Saad MA, Wilson AM (2021). Versatility of hard occlusal splints in optimizing outcomes in patients with old pan-facial fractures. Plast Reconstr Surg Glob Open.

[19] Chun JJ, Yoon SM, Song WJ, Jeong HG, Choi CY, Wee SY (2018). Causes of surgical wound dehiscence: a multicenter study. J Wound Manag Res.

[20] Phillips C, Blakey G 3rd, Essick GK (2011). Sensory retraining: a cognitive behavioral therapy for altered sensation. Atlas Oral Maxillofac Surg Clin North Am.

[21] Hassan AF (2020). Effect of local anesthesia and extraction on mouth opening. Indian J Forensic Med Toxicol.

[22] Krishna BP, Reddy BP, Yashavanth Kumar DS, Ummar M, Shekhar V, Chandra Tiwari RV (2020). Role of serratiopeptidase and dexamethasone in the control of postoperative swelling. Ann Maxillofac Surg.

[23] Nikolajsen L, Minella CE (2009). Acute postoperative pain as a risk factor for chronic pain after surgery. Eur J Pain Suppl.

[24] Eraqi M, Diab AH, Matschke K, Alexiou K (2024). Confirmation of safety of titanium wire in sternotomy closure, a randomized prospective study. Thorac Cardiovasc Surg.

